# The Price of Surviving on Adrenaline: Developmental Programming Responses to Chronic Fetal Hypercatecholaminemia Contribute to Poor Muscle Growth Capacity and Metabolic Dysfunction in IUGR-Born Offspring

**DOI:** 10.3389/fanim.2021.769334

**Published:** 2021-12-09

**Authors:** Rachel L. Gibbs, Dustin T. Yates

**Affiliations:** Stress Physiology Laboratory, Department of Animal Science, University of Nebraska-Lincoln, Lincoln, NE, United States

**Keywords:** adaptive fetal programming, developmental origins of health and disease (DOHAD), fetal growth restriction, intrauterine growth restriction (IUGR), low birthweight, metabolic programming

## Abstract

Maternofetal stress induces fetal programming that restricts skeletal muscle growth capacity and metabolic function, resulting in intrauterine growth restriction (IUGR) of the fetus. This thrifty phenotype aids fetal survival but also yields reduced muscle mass and metabolic dysfunction after birth. Consequently, IUGR-born individuals are at greater lifelong risk for metabolic disorders that reduce quality of life. In livestock, IUGR-born animals exhibit poor growth efficiency and body composition, making these animals more costly and less valuable. Specifically, IUGR-associated programming causes a greater propensity for fat deposition and a reduced capacity for muscle accretion. This, combined with metabolic inefficiency, means that these animals produce less lean meat from greater feed input, require more time on feed to reach market weight, and produce carcasses that are of less quality. Despite the health and economic implications of IUGR pathologies in humans and food animals, knowledge regarding their specific underlying mechanisms is lacking. However, recent data indicate that adaptive programing of adrenergic sensitivity in multiple tissues is a contributing factor in a number of IUGR pathologies including reduced muscle mass, peripheral insulin resistance, and impaired glucose metabolism. This review highlights the findings that support the role for adrenergic programming and how it relates to the lifelong consequences of IUGR, as well as how dysfunctional adrenergic signaling pathways might be effective targets for improving outcomes in IUGR-born offspring.

## INTRODUCTION

Intrauterine growth restriction (IUGR) is the result of fetal developmental programming aimed at increasing the chances of surviving poor intrauterine conditions by promoting thrifty growth and metabolism. Unfortunately, these same developmental changes also diminish metabolic health and quality of life after birth (Hales and Barker, 2001; Wells, 2011). The link between stress-induced IUGR and post-natal metabolic disorders was first described by Barker and Hales, whose epidemiological studies correlated low birthweight with adulthood obesity, type II diabetes, insulin resistance, and hypertension (Hales et al., 1991; Hales and Barker, 1992; Ariouat and Barker, 1993; Barker et al., 1993). Their dogma-establishing discovery, which they termed the Thrifty Phenotype Hypothesis, has been substantiated by thorough subsequent research that collectively estimates IUGR-born individuals to be at 18-fold greater risk for developing clinical metabolic disease by adulthood (reviewed in detail by Crispi et al., 2017; Yates et al., 2018). IUGR affects up to 25% of pregnancies worldwide and is the 2nd leading cause of perinatal mortality and morbidity (Wu et al., 2006; Hoyert and Gregory, 2020; Tesfa et al., 2020). Moreover, the impact of IUGR on the health of surviving offspring can manifest as early as 3 years of age (Iniguez et al., 2006; Milovanovic et al., 2014). Advances in pre-natal and neonatal care for IUGR infants have markedly improved survival rates in IUGR infants, which makes identifying targetable programming mechanisms for improving long-term metabolic health in these individuals an emerging priority (Hoyert and Gregory, 2020).

Low birthweight in livestock due to IUGR is a barrier to the economic sustainability of meat production, as these animals exhibit greater early-life mortality and lifelong performance deficits (Yates et al., 2018). Severe maternofetal stress is linked to high rates of conceptus loss, which not only reduces the number of offspring but also diminishes the dam’s productivity index (Doney et al., 1976; Hansen, 2014). This may result in pre-mature culling of otherwise desirable females, leading to the loss of high-quality genetics and increasing costs associated with replacement females. In addition to pre-natal demise, IUGR often results in offspring that are born live but weak and lack the vigor needed to stand and nurse appropriately, which frequently leads to secondary starvation, hypothermia, and injury (Wu et al., 2006; Reynolds and Caton, 2011; Flinn et al., 2020). The failure of low birthweight newborns to thrive increases total pre-weaning death losses by up to 15% across livestock species (Wu et al., 2006; Flinn et al., 2020). Although perinatal death loss is a concerning animal welfare issue that warrants improvement, the most common and economically relevant outcomes for low birthweight livestock are poor growth performance and metabolic inefficiency. In response to nutrient restriction (typically associated with placental insufficiency), the fetus develops an asymmetrical growth pattern by preferentially supporting vital tissue growth at the expense of peripheral tissues, primarily skeletal muscle (Bell and Greenwood, 2016; Gibbs et al., 2020). In fact, muscle mass in the IUGR fetus may be restricted by over 50% near term due to thrifty programming (Beede et al., 2019). These deficits persist after birth, as IUGR-born offspring continue to exhibit reduced muscle growth capacity, metabolic dysfunction, and poor body composition (i.e., greater fat-to-lean mass ratios) (Cadaret et al., 2019c; Gibbs et al., 2019, 2020; Yates et al., 2019). Consequently, low birthweight animals require more time on feed to reach market weight and produce lighter, less meritorious carcasses with smaller retail cuts and increased fat trim (Robinson et al., 2013; Bell and Greenwood, 2016; Greenwood and Bell, 2019).

The establishment and characterization of several animal models for IUGR provides the opportunity to study the fetal programming mechanisms underlying IUGR pathologies in livestock and humans (Reynolds et al., 2010; Beede et al., 2019). Currently, these mechanisms are not comprehensively understood, but recent findings implicate developmental changes in adrenergic regulation of muscle and other tissues that are relevant to nutrient utilization, metabolic homeostasis, and peripheral tissue growth (Yates et al., 2011, 2019; Cadaret et al., 2019c; Gibbs et al., 2020). In this review, we highlight the evidence for adrenergic adaptations in IUGR tissues and their roles in impaired post-natal muscle growth and metabolic dysfunction.

## THE CONSEQUENCES OF IUGR

### Etiology of IUGR in Livestock and Humans

There is a broad range of causes for IUGR in livestock and humans that result in varying degrees of fetal growth restriction, which have been reviewed in greater detail elsewhere (Greenwood and Cafe, 2007; Beede et al., 2019). In livestock, IUGR is most associated with environmental conditions that produce sustained maternal stress responses or that limit nutrient availability. Such conditions commonly include heat stress events, prolonged cold exposure, elevation-associated hypoxia, drought, overgrazing, inadequate nutrient supplementation, and feed or forage toxicity (Wu et al., 2006; Reynolds et al., 2010; Robinson et al., 2013). IUGR can also result from multifetal pregnancies, especially in species for which multiple births are uncommon (Yates et al., 2018; Flinn et al., 2020). IUGR cases in humans can result from environmental factors but are more frequently related to diet and lifestyle factors that lead to maternal nutrient imbalance, which can impact the fetal nutrient supply as well as the maternofetal endocrine milieu (Barker et al., 1993). Additional causes in humans include substance abuse, chronic or acute illness, and the use of assisted reproductive technologies (Barker, 1990; Woo et al., 2017). When such conditions are present during the critical window for placental development, placental stunting ensues (Cheema et al., 2006; Blasio et al., 2007; Carr et al., 2012; Brown et al., 2016; Zhang et al., 2016; Burton and Jauniaux, 2017). The result is a permanent state of placental insufficiency, whereby the stunted placenta is unable to meet the nutritional requirements of the fetus during its exponential growth phase late in gestation (Limesand et al., 2018; Yates et al., 2018). As described in more detail below, IUGR is in essence the tertiary result of fetal programming responses to this secondary insult of placental insufficiency more so than to the primary maternal stressor. Consequently, fetal outcomes are rather consistent despite the broad range of maternal causes.

### Placental Insufficiency, Poor Intrauterine Conditions, and the Inevitability of IUGR

IUGR is necessitated by the increase in nutrient requirements to support normal fetal growth and the inability of the compromised placenta to meet them. Peak placental development occurs from the mid-1st trimester to the late 2nd trimester, which is from approximately day 30 to 100 of gestation in sheep and day 50 to 90 in humans (Burton and Jauniaux, 2017; Flinn et al., 2020). Most maternal stressors that occur during this critical window shift blood flow (and thus nutrient delivery) away from the gravid uterus (Lang et al., 2000; Limesand et al., 2018). For example, heat stress that increases maternal body temperature by as little as 0.7°C redirects maternal blood flow to the skin and nasal mucosa in order to dissipate heat, resulting in concomitant decreases in blood flow through caruncles and cotyledons (Alexander et al., 1987). When sustained, reduced uterine blood flow results in a smaller placenta with diminished vascular density, increased vascular resistance, and reduced expression of nutrient transporters (Regnault et al., 2002; Wallace et al., 2003; Cheema et al., 2006; Brown et al., 2011; Burton and Jauniaux, 2017; Limesand et al., 2018). Collectively, these and other microanatomical changes create a robust impairment in placental transport capacity.

**FIGURE 1 |:**
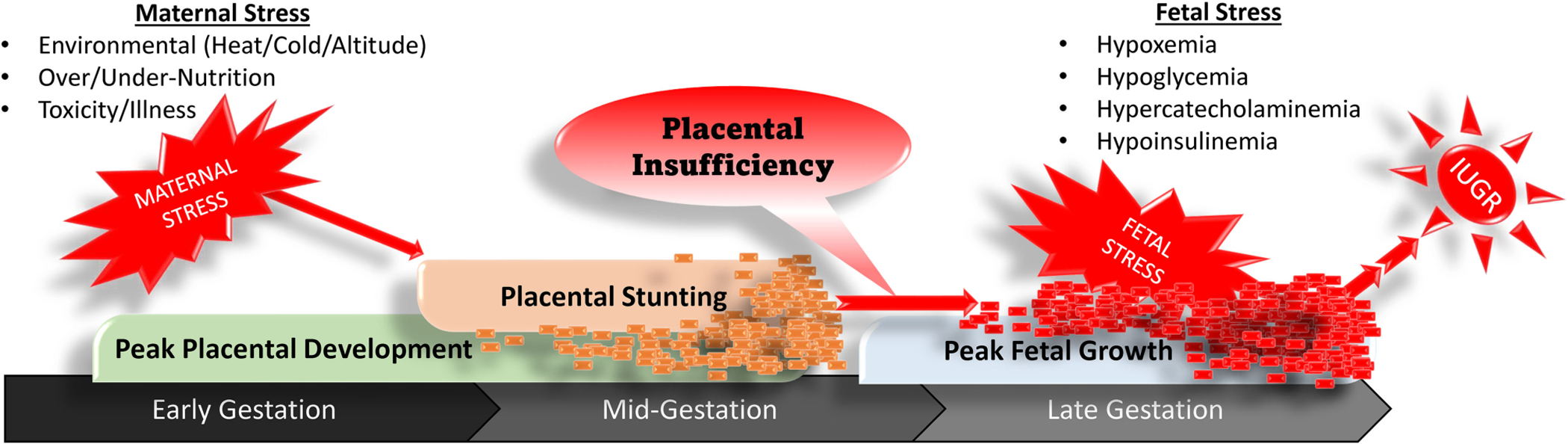
Progression of stress-induced placental stunting in early to mid-gestation that results in placental insufficiency, fetal stress, and intrauterine growth restriction in late gestation.

Placental stunting has little to no effect on the fetus during early and mid-gestation, when its relatively small size creates only modest nutrient demands. However, discrepancies between fetal nutrient requirements and placental capacity begin to appear early in the 3rd trimester and progressively worsen toward term due to rapid fetal growth in late gestation (Yates et al., 2018; Posont and Yates, 2019), as illustrated in Figure 1. The inadequate supply of glucose and O_2_ by the placenta creates chronic fetal hypoxemia and hypoglycemia (Limesand et al., 2007; Macko et al., 2013). The 30–50% reductions in blood O_2_ and glucose initiate fetal stress responses marked by pronounced increases in circulating concentrations of the catecholamines, epinephrine and norepinephrine (Leos et al., 2010; Rozance et al., 2018), in addition to inflammatory cytokines and other endocrine factors (Jones et al., 2018; Cadaret et al., 2019b). Increased adrenergic activity alters nutrient utilization, blood flow, and insulin secretion so as to preferentially redirect glucose and O_2_ that is available to the most essential tissues (Morrison, 2008; Camacho et al., 2017; Rozance et al., 2018; Yates et al., 2019; Davis et al., 2020). Like other nutrients, placental transport of amino acids is also reduced in IUGR pregnancies (Brown et al., 2011). In response, the IUGR fetus slows protein accretion in peripheral tissues substantially, which helps to maintain blood concentrations for most amino acids and provides an additional energy substrate for essential tissues (Rozance et al., 2018). Less is understood about fatty acid fluxes in the IUGR fetus. Recent findings in IUGR baboon fetuses indicate that circulating free fatty acid concentrations near term appear to be normal (Chassen et al., 2020). However, adrenergic activation under non-pathological conditions is known to increase fatty acid mobilization *via* lipolysis (Beard et al., 2018). Similar effects in the IUGR fetus may ultimately diminish fat stores, which would help to explain the well-documented disruption in perinatal thermoregulation (Flinn et al., 2020). It is important to note that developmental responses of the IUGR fetus to intrauterine stress increase its chances for survival and only become problematic after birth, when the absence of chronic stress creates a mismatch with the IUGR-born offspring’s metabolic programming (Boehmer et al., 2017; Limesand and Rozance, 2017; Yates et al., 2019).

### The Tissue-Specific Impacts of IUGR

**FIGURE 2 |:**
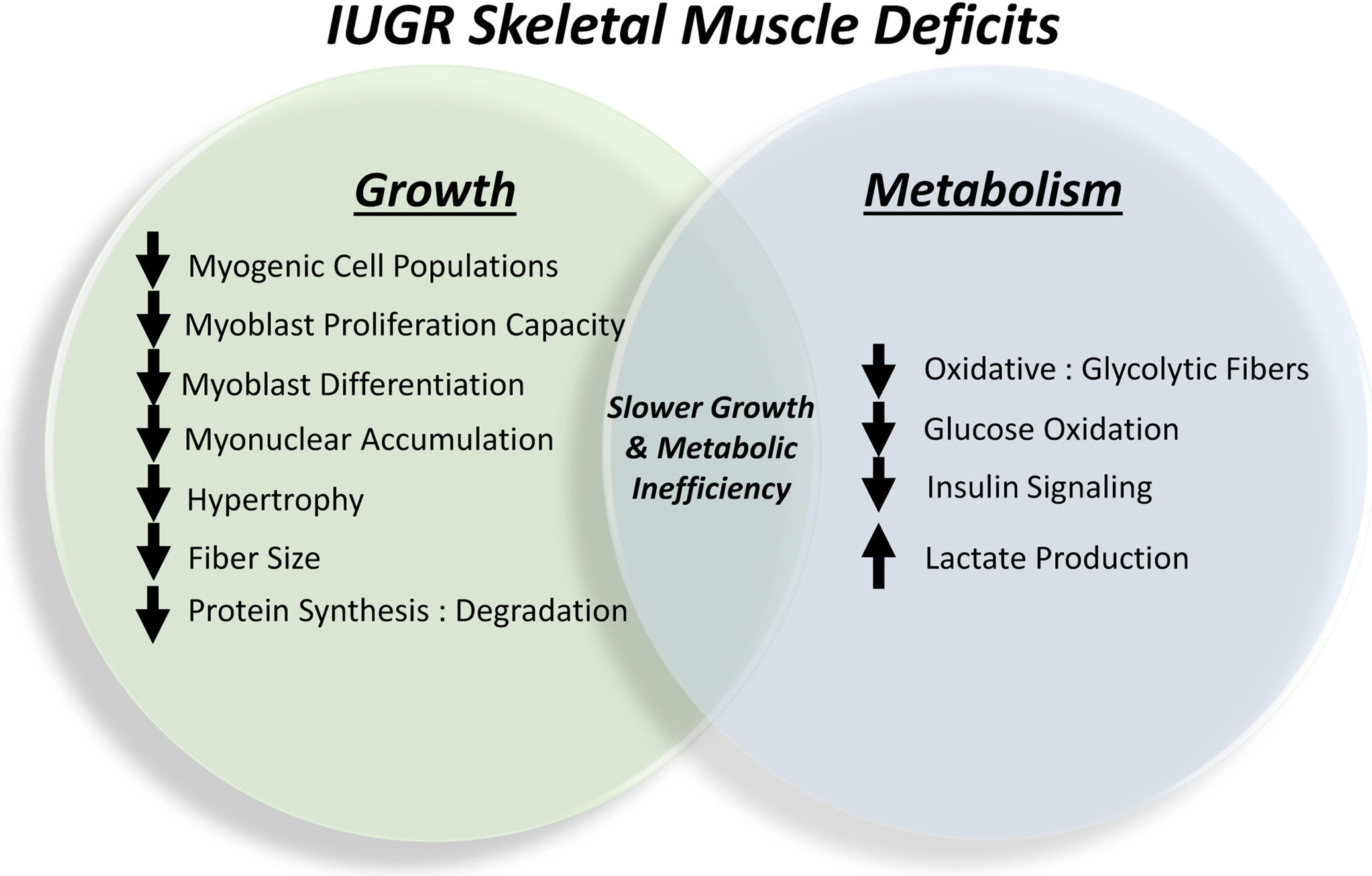
Summary of the outcomes of skeletal muscle programming in the IUGR fetus that contribute to lifelong impairments in growth capacity and metabolic homeostasis.

In response to chronic stress conditions, IUGR fetuses undergo a number of programming adjustments that disproportionally suppress growth and metabolism of peripheral tissue relative to that of bone, neural, and hepatic tissues (Yates et al., 2011, 2019; Macko et al., 2013, 2016; Posont et al., 2018; Cadaret et al., 2019c). The sparing of certain tissues at the expense of others creates the asymmetric growth patterns that are hallmark to IUGR fetuses and offspring. In IUGR fetal, neonatal, and juvenile lambs this has been shown to manifest morphometrically as greater body length-to-bodyweight, brain-to-bodyweight, and liver-to-bodyweight ratios (Cadaret et al., 2019c; Yates et al., 2019; Gibbs et al., 2020). Skeletal muscle is perhaps the most profoundly targeted peripheral tissue due it its high rates of glucose and O_2_ consumption and its sensitivity to adrenergic regulation. In the uncompromised fetus, skeletal muscle accounts for ~65% of total glucose consumption and 85% of insulin-stimulated glucose utilization, and these processes can be interrupted by increased catecholamine concentrations (Brown, 2014). Consequently, adaptive programming restricts skeletal muscle growth capacity and metabolic function (Yates et al., 2012b, 2014; Chang et al., 2019), as summarized in Figure 2. Histological assessments in fetal sheep show that IUGR skeletal muscle fibers are smaller in diameter and contain fewer myonuclei, which limits the capacity for protein synthesis and accretion (Yates et al., 2014, 2016). Reductions in the myonuclear accumulation essential for hypertrophic muscle fiber growth are the result of intrinsic functional impairments in myogenic stem cells known as myoblasts. These cells comprehensively exhibit diminished proliferation capacity (Yates et al., 2014; Soto et al., 2017; Posont et al., 2018) and may also exhibit reduced differentiation (Posont et al., 2021), both of which are rate-limiting steps in the facilitation of muscle fiber growth through fusion-mediated nuclei donation. Impaired myonuclear accumulation and reduced protein synthesis relative to protein degradation lead to reductions in muscle mass (Brown et al., 2011; Yates et al., 2014; Rozance et al., 2018; Chang et al., 2019).

In addition to limiting muscle mass, IUGR fetal adaptations also spare nutrients and O_2_ by directly altering metabolic processes in skeletal muscle (Posont and Yates, 2019; Pendleton et al., 2021). Specifically, less glucose is oxidized by muscle in favor of greater glycolytic lactate production, which provides a substrate source for hepatic gluconeogenesis *via* the Cori cycle. In fetal, neonatal, and juvenile lambs, this manifested in reductions of up to 50% in hindlimb-specific glucose oxidation rates, which were concomitant with increased blood lactate concentrations (Cadaret et al., 2019a,c; Yates et al., 2019; Gibbs et al., 2021; Posont et al., 2021). Similar impairment of glucose oxidation was observed in *ex vivo* assessments of primary skeletal muscle isolated from these animals. Because the majority of glucose is metabolized by skeletal muscle, reduced whole-body glucose oxidation rates were also observed in IUGR fetal sheep (Limesand et al., 2007; Brown et al., 2015). Interestingly, these reductions in muscle glucose oxidation were independent of muscle glucose utilization and insulin sensitivity in most cases. The shift in skeletal muscle metabolism coincided with changes in fiber type proportions, as *semitendinosus* and *biceps femoris* (i.e., upper hindlimb) muscles from near-term IUGR fetal sheep exhibited 20–50% reductions in the proportion of oxidative fibers (i.e., Types I and IIa) relative to glycolytic fibers (i.e., Type IIx) (Yates et al., 2016). Changes in metabolic processes are accompanied by disruption of insulin signaling pathways, as demonstrated by reduced phosphorylation of the insulin signaling hub Akt in primary *flexor digitorum superficialis* muscle from IUGR fetal and neonatal sheep when incubated with low or high insulin concentrations (Cadaret et al., 2019a,c; Yates et al., 2019; Posont et al., 2021). This impairment is almost certainly a contributing factor in the greater frequency of insulin resistance in IUGR-born offspring (Dulloo et al., 2006; Lorenzo et al., 2008; Macko et al., 2013, 2016; Camacho et al., 2017).

Glucose homeostasis in the IUGR fetus and offspring is further impeded by reductions in pancreatic islet mass and function (Boehmer et al., 2017; Camacho et al., 2017; Chen et al., 2017). β cells located within pancreatic islets produce and secrete insulin in response to elevated blood glucose, which in turn stimulates muscle and other insulin-sensitive tissues to clear glucose from circulation for metabolism or storage. However, this function is diminished by IUGR pathologies that reduce islet size, β cell proliferation rates, and development of islet microvasculature (Lee and Hennighausen, 2005; Limesand et al., 2005; Kostromina et al., 2013). These disruptions in development impair islet functionality, leading to reduced insulin content and a poor capacity for glucose-stimulated insulin secretion (Limesand et al., 2006, 2007). As with skeletal muscle, functional deficits in pancreatic islets persist in offspring. Indeed, IUGR-born lambs continued to exhibit reduced islet insulin content and impaired glucose-stimulated insulin secretion as neonates (Cadaret et al., 2019c; Yates et al., 2019).

**FIGURE 3 |:**
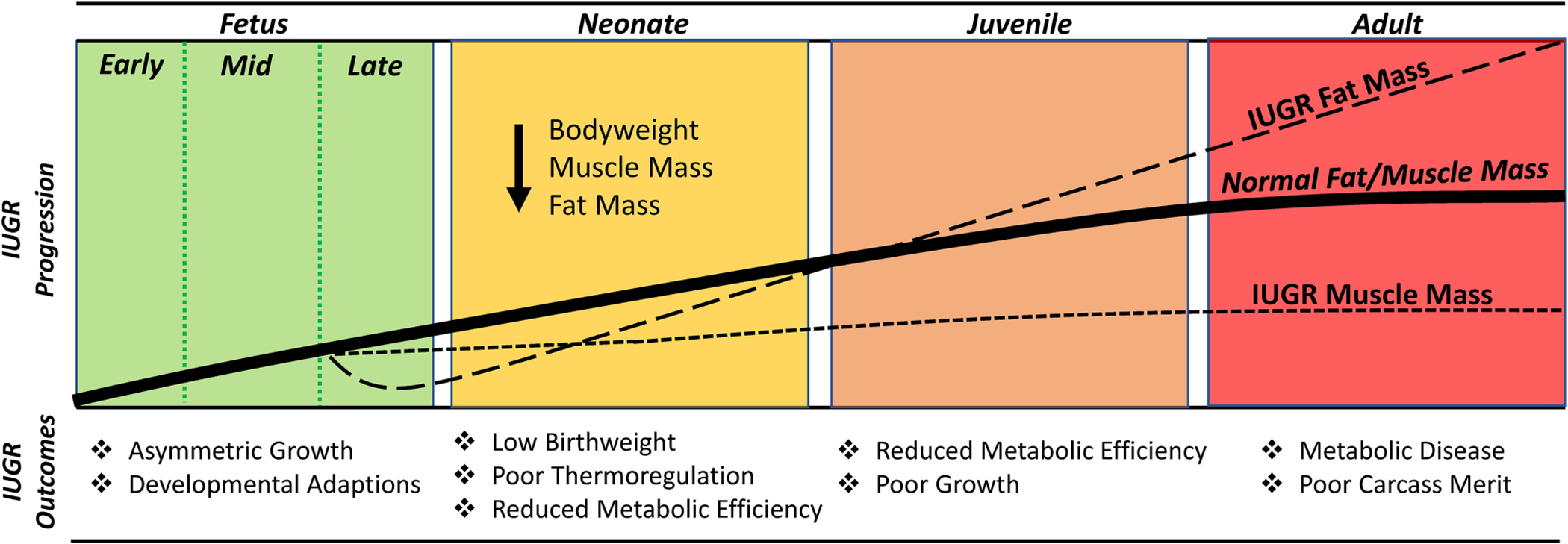
Timeline illustrating the changes in muscle growth capacity and fat deposition in the IUGR fetus/offspring relative to a normal (i.e., uncompromised) fetus/offspring.

Restricted nutrient availability results in a substantial reduction in fat deposition by the IUGR fetus. By the mid-3rd trimester, IUGR fetal sheep exhibited reduced mass of abdominal, pericardial, and hindlimb adipose deposits, which resulted in less whole-body lipid content (Alexander, 1978). Bioelectrical impedance estimates in IUGR-born lambs indicated that fat mass continued to be reduced in the neonatal stage (Gibbs et al., 2019). The lack of fat in IUGR newborns coupled with a 2.5-fold reduction in expression of uncoupling protein 1 (UCP1) helps to explain their reduced capacity for thermoregulation (Flinn et al., 2020). Unlike deficits in skeletal muscle mass, the discrepancies in fat mass begin to wane beyond the neonatal stage, as illustrated in Figure 3. In fact, IUGR-born lambs and children begin to exhibit *greater*-than-normal fat deposition as juveniles (Dulloo et al., 2006; Zinkhan et al., 2018; Gibbs et al., 2020). This process, known as catch-up growth, coincides with enhanced expression of adipogenic promoters including PPARγ, fatty acid synthase, and acetyl-CoA carboxylase α (Desai and Ross, 2011; Zinkhan et al., 2018). The excessive storage of nutrients as fat is a reflection of the mismatch created by thrifty metabolic programming but adequate nutrient availability after birth (Desai and Ross, 2011). Moreover, the greater propensity for fat deposition combined with restricted muscle growth capacity leads to less desirable body composition and carcass merit that is observed in IUGR-born livestock at harvest (Robinson et al., 2013; Bell and Greenwood, 2016; Greenwood and Bell, 2019).

## ADRENERGIC PROGRAMMING IS A MECHANISTIC DRIVER OF IUGR OUTCOMES

### Adrenergic Regulation of Growth and Metabolism

**FIGURE 4 |:**
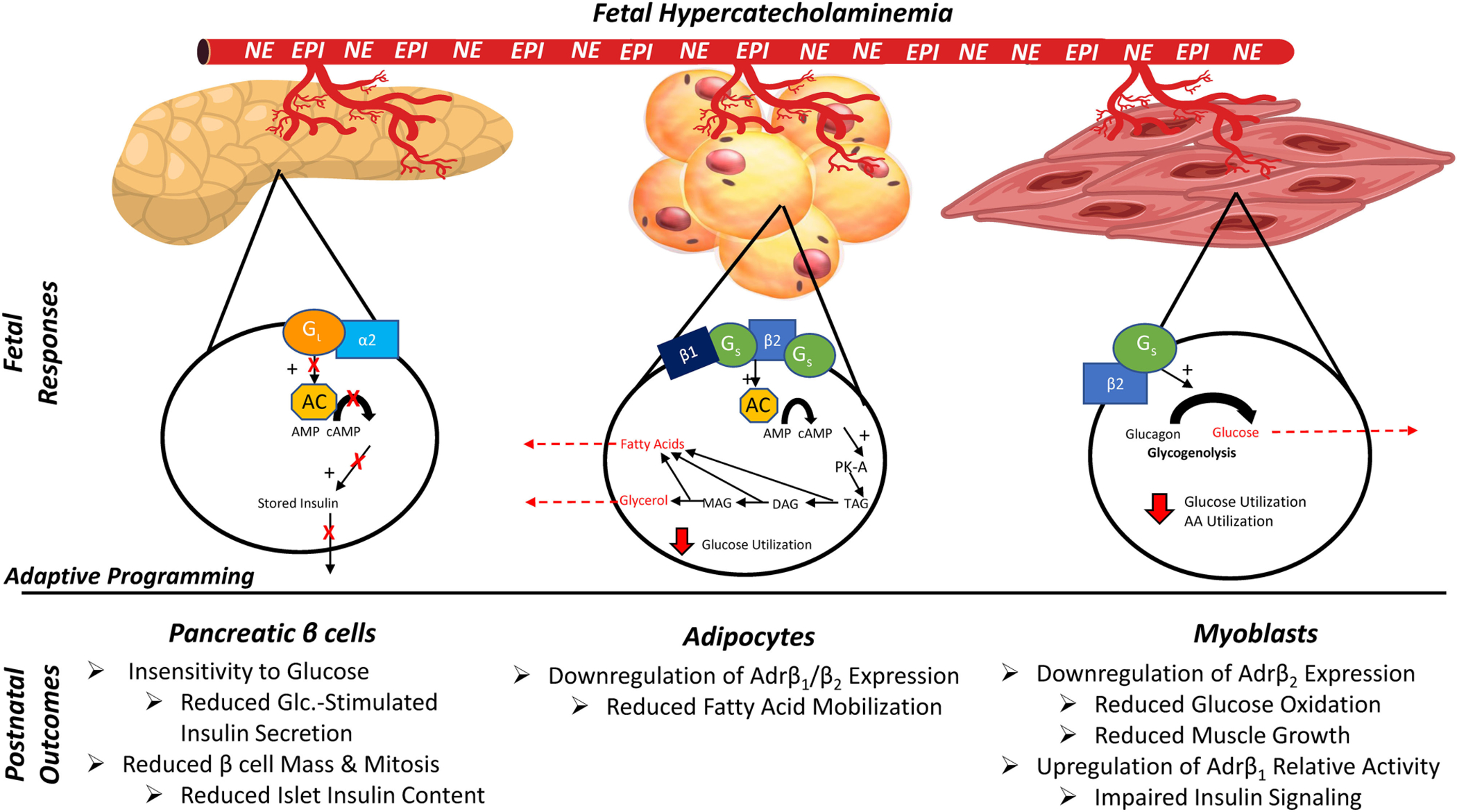
Tissue-specific outcomes of adrenergic programming in the IUGR fetus/offspring due to chronic exposure to elevated circulating catecholamine concentrations *in utero*. EPI, Epinephrine; NE, norepinephrine; G_i_, inhibitory G-protein α subunit; G_s_, stimulatory G-protein α subunit; α2, β1, β2, adrenergic receptors; AC, adenylyl cyclase; cAMP, cyclic AMP; PKA, protein kinase A; TAG/DAG/MAG, tri/di/monoacylglycerol; AA, amino acids.

The adrenergic system is an intricate and robust regulatory system that utilizes multiple signaling pathways to elicit changes associated with stress responses, growth, and metabolism across many tissue types. Epinephrine and norepinephrine are catecholamines that serve as the primary ligands for the adrenergic system (Diego et al., 2008). Although norepinephrine is an important neurotransmitter, almost all catecholamines released into circulation during stress originate from the chromaffin cells of the adrenal medulla (Yates et al., 2012b). Catecholamines act by binding G protein-coupled adrenoceptors on cellular surfaces, which in turn activate 2nd messenger pathways. Adrenoceptors (Adr) exist in nine subtypes among two major classes (α_1A_, α_1B_, α_1D_, α_2A_, α_2B_, α_2C_, β_1_, β_2_, and β_3_), which are expressed in tissue-specific combinations throughout the body (Ciccarelli et al., 2017). As summarized in Figure 4, the tissue specificity of Adr profiles allow intricate regulation of physiological processes associated with growth and development, blood flow, metabolism, and other cellular functions in skeletal muscle, adipose tissue, and pancreatic islets, among other tissue types (Beermann, 2002; Anthony and Henry, 2015; Chen et al., 2017; Beard et al., 2018).

In skeletal muscle, acute stimulation of Adrβ_2_ (the most abundant isoform expressed by muscle tissues) modestly reduces glucose uptake but increases glycogen breakdown, glucose oxidation, and protein synthesis (Claeys et al., 1989; Anthony and Henry, 2015; Cadaret et al., 2017; Kelly et al., 2018). Adrβ_2_ pathways also work additively with insulin to activate Akt *via* phosphorylation and to increase insulin-stimulated glucose oxidation (Bodine et al., 2001; Rommel et al., 2001; Cadaret et al., 2017; Camacho et al., 2017). Sustained stimulation of Adrβ_2_ increases muscle mass and leanness by enhancing myoblast proliferation and myonuclear accumulation in muscle fibers, which in turn increases their capacity for protein synthesis and accretion (Beermann, 2002; Cadaret et al., 2017). Findings in adult muscle indicate that activation of Adrβ_1_ under normal conditions can impede insulin-stimulated Akt phosphorylation, but low expression of this isoform by muscle creates only modest implications for myoblast function, protein synthesis, and metabolism (Yates et al., 2012a; Cadaret et al., 2017). In contrast, adipose tissue contains substantial amounts of Adrβ_1_ and Adrβ_3_ that, when activated, reduce glucose uptake and increase lipolysis and fatty acid mobilization in concert with Adrβ_2_ and hormone sensitive lipase (Lafontan and Langin, 2009). Mobilized free fatty acids can then be utilized for β oxidation by muscle, liver, and other tissues (Anthony and Henry, 2015; Beard et al., 2018). Adrenergic regulation of pancreatic islets, which has been reviewed in great detail elsewhere (Boehmer et al., 2017; Limesand and Rozance, 2017), is somewhat paradoxical. Elevated circulating catecholamine concentrations profoundly inhibit glucose-stimulated insulin secretion, primarily through the activation of Adrα_2C_ and other α isoforms in β cells (Leos et al., 2010; Andrews et al., 2015). However, basal adrenergic activity appears to be essential for proper islet development *in utero*, as adrenal demedullation in otherwise uncompromised fetal sheep diminished insulin secretion (Yates et al., 2012b; Macko et al., 2016).

### Chronic Catecholamine Exposure Alters Adrenergic Programming in IUGR Tissues

In response to progressive hypoxemia and hypoglycemia during late gestation, the IUGR fetus sustains increases of up to 7-fold in circulating catecholamines, which are only alleviated by birth (Leos et al., 2010; Macko et al., 2013, 2016). Brief spikes in fetal catecholamines occur periodically even in uncompromised pregnancies. However, when hypercatecholaminemia is sustained for days or even weeks (as it is in the IUGR fetus), fetal programming responses are induced that alter adrenergic responsiveness in catecholamine-sensitive tissues. Protein and gene expression analyses in skeletal muscle and myoblasts from IUGR fetal and neonatal sheep indicate substantial reductions in Adrβ_2_ but normal expression of Adrβ_1_ (Yates et al., 2018, 2019). This appears to shift the relative adrenergic tone by reducing the effects of stimulatory Adrβ_2_ pathways relative to those of inhibitory Adrβ_1_ pathways. The nature of this change in adrenergic tone is consistent with impaired muscle insulin signaling, myoblast proliferation and differentiation, and insulin-stimulated glucose oxidation (in the absence of impaired glucose uptake) described in earlier sections. Additional work is warranted to more thoroughly characterize the observed adrenergic programming, but existing evidence indicates that it almost certainly contributes to the persistent deficits in muscle mass and glucose oxidation exhibited by IUGR-born offspring (Cadaret et al., 2019c; Gibbs et al., 2019, 2020, 2021; Posont et al., 2021).

Chronic exposure to elevated catecholamines also decreased Adrβ_2_ and increased Adrβ_3_ in IUGR fetal and neonatal adipose tissue, with no apparent effect on Adrβ_1_ expression (Myers et al., 2008; Chen et al., 2010). As in skeletal muscle, the programmed desensitization of Adrβ_2_ expression and function in adipose tissue serves as a compensatory mechanism to partially offset chronic adrenergic stimulation. This adaptation benefits the developing fetus but has metabolic ramifications following birth, when circulating catecholamine concentrations return to normal. Specifically, IUGR neonatal lambs exhibited a 55% reduction in fatty acid mobilization when infused with epinephrine (Chen et al., 2010). Impaired fat mobilization together with the increased propensity for fat storage helps to explain adipose driven catch-up growth typically observed in IUGR-born offspring as they approach the juvenile stage (Dulloo et al., 2006; Lafontan and Langin, 2009; Desai and Ross, 2011; Gibbs et al., 2020).

Adrenergic programming in IUGR pancreatic islets has been comprehensively characterized by the work of SW Limesand, PJ Rozance, and others (Boehmer et al., 2017; Limesand and Rozance, 2017). Their studies have utilized fetal adrenal demedullation, pharmaceutical adrenergic blockade, and direct norepinephrine infusions to demonstrate that elevated circulating catecholamines are the primary inhibitors of insulin secretion in IUGR fetal sheep (Leos et al., 2010; Yates et al., 2012b; Macko et al., 2013, 2016; Chen et al., 2014, 2017). Chronic exposure of fetal islets to catecholamines during late gestation also reduced gene expression for Adrβ_1_, Adrα_1D_, Adrα_2A_ and Adrα_2C_, indicating robust adrenergic insensitivity (Chen et al., 2014, 2017). Because Adrα_2_ pathways inhibit glucose stimulus-secretion coupling in islet β cells (Sperling et al., 1980; Jackson et al., 1993, 2000), fetal glucose-stimulated insulin secretion was actually enhanced (i.e., greater than in uncompromised fetuses) when elevated adrenergic activity was removed or blocked (Leos et al., 2010; Chen et al., 2014, 2017). Moreover, enhanced glucose-stimulated insulin secretion persisted in IUGR newborn lambs (Camacho et al., 2017), which may contribute to the dangerous perinatal hypoglycemia that occurs in low birthweight babies. Nevertheless, enhancement of β cell stimulus-secretion coupling is transient, and glucose-stimulated insulin secretion begins to falter in the late neonatal stage and into adulthood (Thorn et al., 2011; Cadaret et al., 2019c; Yates et al., 2019; Gibbs et al., 2021). This may be due to the intrinsic reductions in Adrβ expression, as β adrenergic activity is necessary for proper islet development (Borden et al., 2013).

### Targeting Adrenergic Adaptations Improves IUGR Outcomes

The identification and characterization of adrenergic programming mechanisms in IUGR tissues provides a target for potential treatment and intervention strategies. Indeed, animal studies have begun to provide the fundamental basis for adrenergic manipulation as a strategy to improve growth and metabolic outcomes in IUGR fetuses and offspring. In IUGR fetal sheep, pharmaceutical blockade of elevated adrenergic activity *via* direct fetal infusion of Adrβ/α antagonists yielded immediate recovery of glucose-stimulated insulin secretion (Leos et al., 2010; Macko et al., 2013). Although fetal infusions are perhaps not a realistic option for livestock or even humans in most cases, these studies provide the basis for strategies that target fetal hypercatecholaminemia in more practical ways. For example, a follow-up study found that inducing normoxia in IUGR fetal sheep *via* maternal O_2_ supplementation also improved glucose-stimulated insulin secretion (Macko et al., 2016). Moreover, intermittent daily maternal hyperoxygenation of ewes carrying IUGR fetuses for the final 2 weeks of gestation improved fetal O_2_ status, which in turn improved their birthweight, post-natal growth and body composition, neonatal insulin secretion, and skeletal muscle glucose oxidation (Cadaret et al., 2019c). Although hypercatecholaminemia is not the only outcome of chronic fetal hypoxemia, it is reasonable to assume that part of the benefit observed with O_2_ supplementation was due to moderation of heightened adrenergic activity. In addition to pre-natal interventions, adrenergic programming may be an effective target for post-natal treatment strategies as well. In IUGR-born neonatal sheep, daily oral administration of the Adrβ_2_ agonist clenbuterol together with the Adrβ_1_ antagonist atenolol and the Adrβ_3_ antagonist SR59230A from birth to 30 days of age improved peripheral tissue insulin sensitivity and enhanced glucose utilization rates (Yates et al., 2019). However, this approach failed to recover deficits in skeletal muscle growth and glucose oxidation or in glucose-stimulated insulin secretion, perhaps indicating that oral administration was ineffective. In a subsequent study, IUGR-born lambs were administered the Adrβ_2_ agonist clenbuterol *via* daily intramuscular injection (rather than by oral bolus). By 60 days of age, these lambs exhibited substantial improvements in growth, muscle mass, and body symmetry (Gibbs et al., 2020). Greater fat deposition was observed in IUGR lambs at 60 days of age that was not observed at 30 days of age, but this too was improved by daily clenbuterol injections. *In vivo* and *ex vivo* metabolic studies showed that daily clenbuterol injections at least partially recovered glucose-stimulated insulin secretion and skeletal muscle glucose oxidation, which was reflected by improvements in early-life whole-body O_2_ consumption rates (Gibbs et al., 2020, 2021).

### The IUGR Phenotype Does Not Result From Adrenergic Programming Alone

The complexity of IUGR programming means that targeting adrenergic dysfunction alone is unlikely to fully recover growth and metabolic deficits in their entirety. For example, adrenal demedullation of IUGR fetal sheep did not improve deficits in pancreatic islet development and only partially corrected insulin secretion (Davis et al., 2015; Macko et al., 2016). Moreover, infusion-induced fetal hypercatecholaminemia in the absence of hypoxia, hypoglycemia, hypoinsulinemia, and other IUGR conditions resulted in less profound impairment of growth and metabolic function (Bassett and Hanson, 1998, 2000; Chen et al., 2014, 2017; Davis et al., 2020, 2021). When maternofetal O_2_ supplementation was used to improve fetal oxemic status in sheep, metabolic improvements exceeded the impact on apparent adrenergic tone. Specifically, acute fetal normoxia improved insulin secretion in the IUGR fetus prior to reductions in circulating norepinephrine (Macko et al., 2016), and daily maternofetal oxygenation of IUGR pregnancies late in gestation improved post-natal skeletal muscle growth and metabolism without recovering Adrβ_2_ content (Cadaret et al., 2019c). Recent studies in sheep and other animal models for IUGR have indicated major roles for inflammatory programming (Cadaret et al., 2019a,b; Beer et al., 2021; Lacey et al., 2021; Posont et al., 2021), glucocorticoid exposure (Miller et al., 2012; Morrison et al., 2012), and poor amino acid balance (Wai et al., 2018; Stremming et al., 2020) in the development of IUGR pathologies. Although manipulation of adrenergic activity was effective in improving some key IUGR pathologies, it is clear that a more comprehensive understanding of the independent and interacting mechanisms that contribute to the IUGR phenotype is necessary to fully recover metabolic health in IUGR-born offspring.

## IMPLICATIONS

Stress-induced fetal programming mechanisms that create the thrifty IUGR phenotype and impair post-natal growth capacity and metabolic function have not been comprehensively characterized, despite the impact of IUGR in humans and livestock. However, recent studies in sheep and other animal models have implicated developmental changes in adrenergic sensitivity of several tissues as a contributing factor for IUGR pathologies. Although additional underlying mechanisms almost certainly exist, the ability of adrenergic programming to be manipulated with well-characterized pharmaceuticals make it an appealing potential target for intervention and treatment strategies. Indeed, the broad availability of isoform-specific adrenoceptor agonists and antagonists could provide a variety of options for therapeutic strategies to improve outcomes in IUGR-born offspring. Low birthweight due to IUGR remains a global health issue and barrier to sustainable meat animal production that will only worsen with the emergence of climate change. Realistic options for improving lifelong metabolic health in IUGR-born individuals and for recovering growth performance and efficiency in livestock are needed, and growing evidence indicates that strategies built around the correction of adrenergic tissue regulation may be an effective approach.
